# Comparative Evaluation of Assumption Lean Community Detection Methods for Human Connectome Networks

**DOI:** 10.1101/2025.11.13.688333

**Published:** 2025-11-14

**Authors:** Ayoushman Bhattacharya, Nilanjan Chakraborty, Xintian Wang, Jiaxin (Cindy) Tu, Donna Dierker, Andy Eck, Soumen Lahiri, Adam Eggebrecht, Muriah D. Wheelock

**Affiliations:** 1 Department of Statistics and Data Science, Washington University in St. Louis.; 2 Mallinckrodt Institute of Radiology, Washington University in St. Louis.; 3 Department of Mathematics and Statistics, Missouri University of Science and Technology.

**Keywords:** Weighted Stochastic Block Model, Infants, Functional Connectivity, Variational Bayes, Community detection, Brain Networks

## Abstract

Community detection provides a principled lens on mesoscale organization in functional brain networks, yet many widely used methods presume assortative structure and depend on arbitrary thresholding, which complicates the selection of the community count K. We conducted a systematic benchmark of three assumption lean approaches that operate directly on weighted functional connectivity matrices: the Weighted Stochastic Block Model, Spectral Clustering, and K-means. Performance was assessed on synthetic networks with known ground truth and on three neuroimaging cohorts spanning development, namely the Human Connectome Project, Washington University 120, and the Baby Connectome Project. We compared strategies for choosing K, including post hoc indices such as silhouette, Calinski-Harabasz, C index, modularity, variation of information, Normalized Mutual Information, and zRand, together with a likelihood-based criterion for the Weighted Stochastic Block Model that uses bootstrap confidence intervals for differences in log likelihood between successive values of K. In simulations all methods recovered stable partitions, but the post hoc indices favored incorrect values of K under weak signal and nonassortative mixing. In adult datasets the indices do not yield a unique optimum, whereas the likelihood-based criterion selects a parsimonious range centered near K=11, which is consistent with established sensory and association systems. In infants and toddlers, the same procedure supports a larger K around 15 and reveals developmentally distinct mesoscale architecture, including anterior and posterior subdivisions within default mode and fronto parietal systems. A consensus relabeling scheme based on Hungarian matching with Hamming distance further stabilizes solutions across runs and across values of K. Overall, threshold free weighted methods mitigate assortative bias and the likelihood-based comparison provides a reproducible path to selecting K.

## Introduction

The human brain is an intricate network made up of neurons and neuronal populations, with numerous regions continually interacting during both resting states and active cognitive processes. Understanding how these regions organize into functional communities is essential for interpreting large-scale patterns of neural communication. Although a wide range of algorithms has been used to identify brain communities in adults, recent work has begun applying community detection methods to the developing brain as well. However, despite this progress, existing studies provide limited guidance on how to determine the optimal number of communities in practice. This gap poses a significant challenge for real-life neuroimaging datasets, where developmental changes, noise, and inter-subject variability complicate community estimation.

Clustering algorithms widely used in the literature often make strong assumptions about brain structure. While there is a broad consensus among neuroscientists that the human connectome is predominantly assortative, research has demonstrated that many but not all communities are assortative (Betzel et al., 2018). The most commonly used community detection algorithms in the neuroimaging literature, namely modularity maximization (Blondel et al., 2008; Newman & Girvan, 2004), Infomap (Rosvall & Bergstrom, 2008), von Mises-Fisher distribution (Yeo et al., 2011), and gradient-weighted Markov Random Field model (Schaefer et al., 2018), require that sub-networks to be internally dense and externally sparse. This requirement can constrain the connectome’s meso-scale structure to be strictly assortative (Betzel, 2020; Betzel et al., 2018; Faskowitz & Sporns, 2020). In contrast, community detection algorithms like the Weighted Stochastic Block Model (WSBM), Spectral Clustering, and K-means, do not require networks to be assortative and can effectively detect communities even in non-assortative networks (Betzel et al., 2018).

The determination of the optimal number of communities remains an open challenge in the literature for several reasons. Algorithms like Infomap detect communities by following the trajectory of a random walker and generate multiple partitions by scanning across different edge densities. Similarly, modularity maximization algorithm seeks to optimize the quality of the network partition by maximizing the modularity of the thresholded connectivity matrix. This approach focuses on preserving higher connection probabilities compared to those predicted by a null model, which typically involves comparing the observed network against randomly generated networks, such as the Newman-Girvan model. However, thresholding the connectivity matrix can lead to a significant loss of information (Aicher et al., 2015). For both Infomap and modularity maximization, the number of detected communities depends heavily on the sparsity threshold: sparser thresholds yield more communities, while denser thresholds produce fewer. Moreover, modularity maximization suffers from the resolution limit problem, which hinders its ability to detect smaller communities. Conversely, other approaches, such as the asymptotical surprise algorithm (Nicolini et al., 2017), often identify extremely small communities that may not be biologically meaningful. Several existing works have also used post hoc measures such as Normalized Mutual Information (NMI), stability analysis (Yeo et al., 2011), the zRand index (Akiki & Abdallah, 2019), VI distance (Faskowitz & Sporns, 2020), and the silhouette coefficient to determine the optimal number of communities. However, different algorithms often produce different number of communities or community assignments based on these posthoc measures (Sanchez-Rodriguez et al., 2021; Yeo et al., 2011) thus making it challenging to determine the ground truth community structure in brain connectome datasets

Stochastic Block Model (SBM) and WSBM are well studied in network analysis, with applications in social, protein, and gene networks. More recently, the WSBM, introduced by Aicher et al. (2015), has been applied in network neuroscience to detect communities in brain networks and other organisms. Studies have demonstrated that communities derived using WSBM show greater hemispheric symmetry and are more widely distributed across the brain, often spanning distant cortical and subcortical regions while still reflecting coherent connectivity patterns compared to those identified by modularity maximization (Betzel et al., 2018; Faskowitz et al., 2018; Faskowitz & Sporns, 2020; Tooley et al., 2022). Additionally, from a neuroscience perspective, where an assumption lean algorithm is preferred for practical purposes, WSBM does not impose rigid assumptions on community structure, unlike modularity maximization and Infomap. This flexibility allows WSBM to estimate a range of network structures, including modular, disassortative, and core-periphery configurations (see Betzel et al., 2018). Along with WSBM, two other assumption lean clustering methods that are widely studied in the statistics literature are Spectral Clustering and K-means. Spectral Clustering has also been widely applied in neuroscience, including to identify functional states in fMRI studies (Abbe, 2018; Cribben & Yu, 2017; Rohe et al., 2011). K-means, known for its computational efficiency, has been used in PET and fMRI studies of the adult brain and has successfully recovered central and peripheral brain communities (Thirion et al., 2014; Tononi et al., 1998). Although WSBM has been implemented for identifying the number of communities in the rat brain (Faskowitz & Sporns, 2020) and adolescent datasets (Tooley et al., 2022). WSBM and other assumption lean clustering methods have not been applied to younger children within the first three years of life.

In this article, we address existing gaps by evaluating three community detection algorithms that make minimal assumptions about brain network structure: WSBM, Spectral Clustering, and K-means. Our goal is to determine the optimal number of communities without relying on thresholding or restrictive structural assumptions. We rigorously benchmark these methods using synthetic and adult datasets, where the true or evidence-based number of communities has been previously modeled with the Infomap algorithm (Gordon et al., 2016), and further evaluate them with post hoc measures including NMI, stability analysis (Yeo et al., 2011), zRand Index (Akiki & Abdallah, 2019), VI distance, and Bayes factors derived from negative log-likelihood (Faskowitz & Sporns, 2020). Finally, we benchmarked these algorithms in the Baby Connectome Project (BCP) dataset.

## Methods

### Human Connectome Project Dataset

The study investigated resting-state fMRI data from the Human Connectome Project (HCP S1200), which is publicly available (Glasser et al., 2013). The HCP study design involved recruiting twins and their family members and conducting two 30-minute resting-state fMRI scans on different days. High-resolution T1-weighted images were obtained using a magnetization-prepared rapid acquisition gradient-echo (MP-RAGE) sequence, with a repetition time (TR) of 2.4 seconds and voxel dimensions of 0.7 × 0.7 × 0.7 mm. Additionally, BOLD contrast-sensitive images were collected using a gradient echo echo-planar imaging (EPI) sequence, featuring a multiband factor of 8, a TR of 0.72 seconds, and voxel dimensions of 2 × 2 × 2 mm. These imaging procedures were conducted on each participant with a custom Siemens SKYRA 3.0T MRI scanner, which was equipped with a specialized 32-channel Head Matrix Coil designed for optimal signal acquisition. To enhance the robustness of the data, the HCP utilized imaging sequences with both left-to-right and right-to-left phase encoding directions. Each participant underwent one imaging session in each direction over two consecutive days (Van Essen et al., 2012). The analysis included 965 healthy adults aged 22 to 35 who retained at least 10 minutes of low-motion data for both Rest 1 and Rest 2 scans after using previously described preprocessing methods including 36 parameters motion regression and motion censoring with frame displacement (FD) exceeding 0.2 mm (Li et al., 2024). Data from the first day of scanning are included in the present analyses.

### Washington University 120 Dataset

The study investigated the Washington University 120 (WU 120) dataset which has been described in prior research (Power et al., 2017). The WU 120 study design included 120 healthy young adults during a relaxed eyes-open fixation task, comprising 60 females aged between 19–32 years with a mean age of 25 years. All participants were native English speakers and right-handed. They were recruited from the Washington University community and screened via a self-report questionnaire to ensure they had no current or past neurological or psychiatric conditions, as well as no head injuries resulting in a loss of consciousness for more than five minutes. Informed consent was obtained from all participants, and the study received approval from the Human Studies Committee and Institutional Review Board at Washington University School of Medicine.

Structural and functional MRI data were obtained using a Siemens MAGNETOM Trio Tim 3T Scanner (Erlangen, Germany) along with a Siemens 12-channel Head Matrix Coil. A T1-weighted sagittal magnetization-prepared rapid acquisition gradient-echo (MP-RAGE) structural image was captured with the following parameters: time echo (TE) of 3.08 ms, time repetition (TR) of 2.4 s, time to inversion (TI) of 1000 ms, a flip angle of 8°, and 176 slices with 1 × 1 × 1 mm voxel sizes. To ensure accurate alignment, an auto-align pulse sequence protocol provided by Siemens was used to position the functional scan slices parallel to the anterior commissure–posterior commissure plane of the MP-RAGE, aligning with the Talairach atlas (Talairach & Tournoux, 1988). During the functional MRI acquisition, participants were instructed to relax and focus on a black crosshair against a white background. The BOLD contrast-sensitive gradient-echo echo-planar imaging (EPI) sequence was employed, with a TE of 27 ms, a flip angle of 90°, and an in-plane resolution of 4 × 4 mm. Whole-brain EPI volumes, consisting of 32 contiguous 4-mm-thick axial slices, were collected every 2.5 seconds. Additionally, a T2-weighted turbo spin-echo structural image (TE = 84 ms, TR = 6.8 s, 32 slices with 1 × 1 × 4 mm voxels) was obtained in the same anatomical planes as the BOLD images to improve atlas alignment. Anterior→Posterior (AP) phase encoding was utilized for the fMRI acquisition, with the number of volumes collected from subjects ranging from 184 to 724 (mean = 336 frames, approximately 14.0 minutes).

Functional images were initially preprocessed to reduce artifacts through the following steps: (1) correction of intensity differences between odd and even slices caused by interleaved acquisition without gaps; (2) realignment to correct for head motion within and across functional runs; and (3) intensity normalization across runs, scaling each to a whole-brain mode value of 1000. Spatial normalization to a standard atlas space was performed for each participant using their corresponding MP-RAGE anatomical scan. Functional data were then resampled into a 3-mm isotropic voxel grid in Talairach space (Talairach & Tournoux, 1988). with motion correction and atlas transformation applied concurrently using a single cubic spline interpolation (Lancaster et al., 1995; Tu et al., 2024) for further details on preprocessing.

### Baby Connectome Project Dataset

Full-term infants (gestational age of 37–42 weeks) without any significant complications during pregnancy or delivery were recruited for the Baby Connectome Project (Howell et al., 2019). All procedures were approved by the Institutional Review Boards of the University of North Carolina at Chapel Hill and the University of Minnesota. Informed consent was obtained from the parents of all participants. MRI images were acquired using a Siemens 3T Prisma scanner with a 32-channel head coil at both the University of Minnesota and the University of North Carolina at Chapel Hill, while the infants were naturally asleep and without the use of sedative medications. The imaging protocols included T1-weighted scans (TR=2400 ms, TE=2.24 ms, 0.8 mm isotropic; flip angle = 8°), T2-weighted scans (TR=3200 ms, TE=564 ms, 0.8 mm isotropic), spin echo field maps (SEFM) (TR=8000 ms, TE=66 ms, 2 mm isotropic, MB=1), and fMRI data (TR=800 ms, TE=37 ms, 2 mm isotropic, MB=8). For fMRI acquisition, a combination of Anterior→Posterior (AP) and Posterior→Anterior (PA) phase encoding directions was utilized, which were then combined into a single time series. An early subset of data was collected with a TR of 720 ms (N = 95). After conducting quality control and preprocessing using previously described methods including 30 parameter motion regression and motion censoring of volumes exceeding 0.2mm filtered FD (Kaplan et al., 2022), the final cohort included 313 fMRI sessions from 181 individuals (95 females, aged 8 to 60 months, with a mean age of 19.1 months and a standard deviation of 8.3 months). The number of low-motion volumes obtained from participants ranged from 840 to 2100, with a mean of 1306 frames (approximately 16.9 minutes).

### Synthetic Network Generation

**Figure 1:**
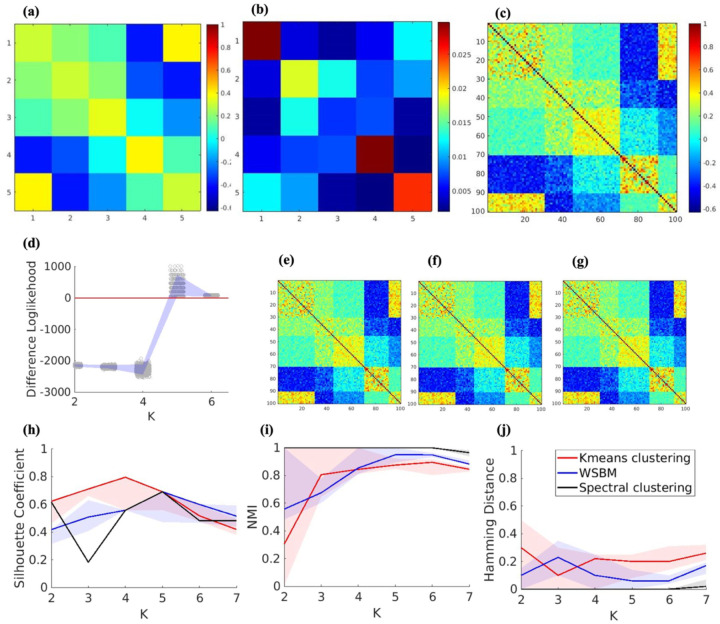
**(a)** Average community connectivity matrix. **(b)** Variability across the community. **(c)** Simulated functional connectivity matrix. **(d)** Bootstrap log-likelihood difference for WSBM. **(e)** Fitted community levels using WSBM with K=5. **(f)** Fitted community levels using Spectral Clustering with K=5. **(g)** Fitted community levels using K-means Clustering with K=5. **(h)** Silhouette coefficient for 1000 replications. **(i)** NMI values for 1000 replications. **(j)** Hamming distance for 1000 replications.

Synthetic brain functional connectivity networks with a pre-specified network count and size were generated for testing the ability of different clustering models to identify the correct number of communities. Using a known probability distribution and a set of controllable parameters, we generated the synthetic networks based on the properties observed in HCP data. Specifically, we created a functional connectivity matrix for 100 Regions of Interest (ROIs) and assigned each randomly to one of the five communities with sizes of 30, 15, 25, 20, and 10 ROIs, respectively. To mimic a human brain connectome, we used the Gordon networks (Gordon et al., 2016). The average connectivity within and between the communities of the matrix were identified from a range of means observed in HCP data (Fig. 1.a). We further calculated the variability of connections within the HCP dataset and obtained a connection variability matrix (Fig. 1.b). After obtaining the average connectivity and variability matrix, we assigned the elements of these matrices to the corresponding pairs of communities. Specifically, a synthetic network with 100 ROIs was generated by incorporating the community structure, average connectivity matrix (Fig. 1.a), and variance connectivity matrix described above (Fig. 1.b) as the ground truth. For each pair of ROIs, the temporal correlation value was drawn from a normal distribution defined by the corresponding mean and variability values from these matrices which leads to the synthetic network in Fig. 1.c.

### Statistical Analysis

Three community detection methods were explored and compared: (1) WSBM, (2) Spectral Clustering (SC), and (3) K-means Clustering. These algorithms were chosen because they make no structural assumptions about the brain networks and do not require thresholding of the functional connectivity (FC) matrices.

### Weighted Stochastic Block Model

The WSBM is one of the most widely used community detection algorithms in machine learning and statistics (Aicher et al., 2015), particularly for identifying community structures within networks. In the WSBM, the vertices of the network are partitioned into K mutually exclusive and exhaustive communities, with each vertex assigned to exactly one of these K groups. Vertices within the same community exhibit similar connectivity patterns. In the case of fMRI data, we treat the brain regions of interest (ROIs) as vertices, and the temporal correlations between these ROIs serve as the edge weights (Gordon et al., 2016; Seitzman et al., 2020).

Mathematically, we denote the n×n FC-matrix as A for n brain ROIs with entries $A_{ij}$ (edge weights) indicating the temporal correlation between vertices i and j. We denote, $z_{i}$ as the membership indicator of i-th vertex and the corresponding probability of it belonging to a particular community (k). The community assignments are assumed to be random, independently and identically distributed with probability distribution $P\left(z_{i}=k\right)=\lambda_{k}$, for all 1≤k≤K and 1≤i≤n. Since the community assignments are mutually exclusive and exhaustive, we assume that $\sum_{k}\lambda_{k}=1$. In the context of neuroscience, we assume that the edge weights follow a normal distribution with community-specific parameters. Under this assumption, the likelihood of the data conditioned on the parameters can be written as follows:

(1) $$P(A|z,\mu ,\Sigma )=\prod_{1\leq i<j\leq n}N\left(A_{ij}|\mu_{z_{i}z_{j}},\sigma_{z_{i}z_{j}}^{2}\right),$$

where $N\left(A_{ij}|\mu_{z_{i}z_{j}},\sigma_{z_{i}z_{j}}^{2}\right)$ denotes the density of a normal distribution with mean $\mu_{z_{i}z_{j}}\in R$ and connectivity variance $\sigma_{z_{i}z_{j}}>0$. The matrix $\mu_{K\times K}=\left(\left(\mu_{ab}\right)\right)$ is the mean connectivity of the group interaction and $\Sigma_{\text{K}\times \text{K}}=\left(\left(\sigma_{ab}^{2}\right)\right)$ is the variability in connection of the group interaction. We assume that the weighted adjacency matrix A is symmetric, i.e., $A_{ij}=A_{ji}$ for all 1≤i<j≤n and $A_{ii}=1$ which we disregard in our analysis since there is no randomness involved in estimating the diagonal elements. We fit $z_{i}$ with flat prior $\pi \left(z_{i}\right)=\frac{1}{k}$ for all 1≤i≤n. For the canonical parameter vector, ${\eta ={\left(\eta_{ab}\right)}^{T}}_{1\leq a,b\leq k}$ with $\eta_{ab}={\left(\frac{\mu_{ab}}{\sigma_{ab}^{2}},-\frac{1}{\sigma_{ab}^{2}},-\frac{\mu_{ab}^{2}}{\sigma_{ab}^{2}}\right)}^{T}$, we assume the conjugate prior from an exponential family taking the form,

(2) $$\pi \left(\eta \right)=\frac{1}{Z\left(\tau \right)}\prod_{1\leq a,b\leq k}\text{exp}\left\{\eta^{T}\tau \right\},$$

where τ is the vector for hyper-parameters and Z(τ) is the normalized constant. Assuming the independence of the priors, we finally have the joint prior as follows,

(3) $$\pi \left(z,\eta \right)=\prod_{1\leq i\leq n}\pi \left(z_{i}\right)\frac{1}{Z\left(\tau \right)}\prod_{1\leq a,b\leq k}\text{exp}\left\{\eta^{T}\tau \right\}.$$

Therefore, the log-likelihood of the joint pdf can be written as

(4) $$l\left(A,z,\eta \right)=\text{log}\;P\left(A|z,\mu ,\Sigma \right)+\text{log}\;\pi \left(z,\eta \right).$$

### Estimation of the model parameters for WSBM

A straightforward maximization of (4) is not feasible because the community memberships are unknown. To address this, we use the Variational Bayes technique (Aicher et al., 2013) to estimate the community memberships $\left(z_{i}\right)$ and the community-specific parameters (μ,Σ). The goal is to approximate the joint posterior distribution of (z,μ,Σ) by minimizing the Kullback-Leibler (KL) divergence between the true posterior distribution and the product of the marginal posterior distributions of z and (μ,Σ).

### Choosing the number of communities

The WSBM implementation assumes that the number of communities K is known apriori. However, in many real-world applications, the value of K is unknown. To estimate K, the Bayes factor based on the log-likelihood is utilized (Aicher et al., 2013). Let $M_{1}$ be the model containing K communities and $M_{2}$ be the model with K+1 communities. We define the Bayes factor as,

(5) $$\text{log}\;B\left(M_{1},M_{2}\right)=\text{log}\frac{P\left(A|M_{1}\right)}{P\left(A|M_{2}\right)}.$$

We constructed bootstrap confidence intervals for the confidence intervals for the Bayes factor for multiple choices of K. The transition at which 95% bootstrap confidence intervals of the Bayes factor overlap with the null value 0 is considered as the optimal choice for K (Aicher et al., 2015; Faskowitz & Sporns, 2020). To this end, we implement the following algorithm to detect the optimal number of communities:

- Step 1: Fit WSBM to the given data 1000 times for a particular value of K. Since the number of unique communities may differ from the input K (Faskowitz & Sporns, 2020), we only keep the valid community assignments (the number of communities in the solution is K).
- Step 2: Repeat step 1 for 2≤K≤20.
- Step 3: For each valid community assignments, bootstrap the difference between log-likelihood values for K and (K+1) 2000 times.
- Step 4: The minimum value of K at which the 95% confidence interval for the differences overlapped with 0 is taken as the optimal number of communities. Specifically, the smallest value of k for which the 95% bootstrap confidence interval of the difference in log-likelihood values between K and K+1 includes 0 is the stopping criterion.

After detecting the optimal number of communities using the above-described algorithm, the final solution is chosen to be the one with maximum-log-likelihood for the optimal K.

### K-means Clustering

K-means clustering is one of the most widely used clustering algorithms in machine learning, statistics, and various other disciplines (Duda et al., 2012; Hastie et al., 2009). It can also be applied to community detection, where the clusters are treated as communities. Given the number of communities, the K-means algorithm identifies the K cluster centroids and assigns each data point to the community corresponding to the closest centroid. In the context of fMRI studies, we use correlation as the dissimilarity measure to determine the proximity of data points to the centroids. We define the dissimilarity measure $d_{ij}=1-r_{ij}$, where $r_{ij}$ is the Pearson correlation coefficient between $i^{\text{th}}$ and $j^{\text{th}}$ rows of the FC matrix and the transformation is taken to make the measure non-negative. Let $y_{i}$ denote the $i^{\text{th}}$ row of the weighted adjacency matrix A for 1≤i≤n. Thus, each $y_{i}$ is a n-dimensional vector. The algorithm is described in steps as follows:

- Step 1: Randomly assign K centroids: $\mu_{1},\ldots ,\mu_{K}$.
- Step 2: Classify n data points $y_{1},\ldots ,y_{n}$ by the closest mean.
- Step 3: Recompute the cluster centroids based on the cluster formed in step 2.
- Step 4: Repeat steps 2–3 until there is no change in the centroids.

To apply the K-means algorithm a prior information on the number of clusters is needed. A variety of post hoc criteria can be used to identify the optimal number of communities, as described in a later section.

### Spectral Clustering

Spectral clustering is a non-parametric clustering algorithm often used in machine learning (Hastie et al., 2009; Newman, 2006). The method has also been used to detect community structure in network data (Abbe, 2018; Rohe et al., 2011). Spectral clustering is found to be useful non-linear structure of data as it tries to preform clustering on the eigen space of the graph Laplacian matrix. For a similarity matrix $S_{n\times n}={\left(\left(S_{ij}\right)\right)}_{1\leq i,j\leq n}$ and $S_{ij}=\text{exp}\left(-d_{ij}^{2}\right)$ denotes the similarity between vertex i and vertex j. We define $d_{ij}$ as the dissimilarity based on the correlation between vertices i and j as earlier. Thus, we formally define the graph Laplacian matrix as L=D-S, where D is a diagonal matrix with entries $D_{ii}=\sum_{1\leq j\leq n;j\neq i}\;S_{ij}$. The algorithm for spectral clustering is described as follows:

- Step 1: Find the eigenvectors $X_{1},\ldots ,X_{K}\in R^{n}$ corresponding to the K eigenvalues of L that are smallest in terms of their absolute values.
- Step 2: Form the matrix $X=\left[X_{1},\ldots ,X_{K}\right]\in R^{n\times K}$ by putting the eigenvectors into the columns.
- Step 3: Treating each of the n rows in X as a point in $R^{K}$ and run the K-means algorithm with K clusters, which creates K nonoverlapping sets $C_{1},\ldots ,C_{K}$ whose union is 1,…,n.
- Step 4: Return the communities $C_{1},\ldots ,C_{K}$. This means that vertex i is assigned to the cluster g if the $i^{\text{th}}$ row of X is assigned to $C_{g}$ in step 3.

Similar to K-means algorithm prior information on the number of communities (K) is required to implement spectral clustering for a real-life data set.

The following table shows the comparison of three algorithms in terms of runtime. Spectral clustering requires an additional $O\left(n^{2}\right)$ time to store the dissimilarity matrix. But such computation is only needed once even for multiple independent iterations.

Table 1 suggests that spectral clustering and K-means clustering are much faster than WSBM in dense networks.

### Post hoc measures for optimal number of community detection

As mentioned earlier, the algorithms discussed in the previous section require prior knowledge of the number of communities or clusters K to begin with. However, such information is typically not available for real-world datasets. To address this, it is common practice to run the algorithms for different values of K and evaluate the quality of the resulting communities using various metrics (Sanchez-Rodriguez et al., 2021). The value of K that yields the best performance based on these metrics is then selected as the solution. However, it is important to note that different metrics may lead to different choices of K. In this section, we will discuss some of the commonly used partition quality metrics in the literature.

We use the following notations to define the metrices. We denote A as the weighted adjacency matrix of the network with n vertices. Let, $d_{ij}$ be the distance/dissimilarity between vertices i and j for 1≤i,j≤n is known. We also define $C=\left\{C_{1},\ldots C_{K}\right\}$ as the K communities and $n_{k}$ be the size of $k^{\text{th}}$ community. We denote $z_{i}$ as the community label for any vertex 1≤i≤n.

### Silhouette Coefficient

Silhouette coefficient is one most commonly used cluster validation (Kaufman & Rousseeuw, 2009). We define the average distance for each vertex $i\in C_{k}$ to every other vertex in the same community as, $a_{i}=\frac{1}{n_{k}-1}\sum_{j\in \left\{C_{k}\setminus i\right\}}\;d_{ij}$ and the minimum average distance to other communities as, $b_{i}=\underset{s\neq k}{\text{min}}\frac{1}{n_{s}}\sum_{j\in C_{s}}d_{ij}$. Thus, the silhouette coefficient is defined as

(6) $$S(k)=\frac{1}{n}\sum_{1\leq i\leq n}\frac{b_{i}-a_{i}}{\text{max}\left\{a_{i},b_{i}\right\}}.$$

The value of silhouette coefficient lies between [−1, 1]. A high value of the coefficient indicates that the objects are well matched to their own clusters and poorly matched with neighbors.

### Modularity

Modularity (Newman, 2004) measures how cohesively the vertices of a network are grouped together, and is defined as

(7) $$Q=\frac{1}{2m}\sum_{1\leq i,j\leq n}\left(A_{ij}-\frac{d_{i}d_{j}}{2m}\right)1\left(z_{i}=z_{j}\right),$$

where $m=\frac{1}{2}\sum_{1\leq i,j\leq n}\;A_{ij}$ is the average edge-weights, $d_{i}=\sum_{1\leq j\leq n;j\neq i}\;A_{ij}$ denotes the degree of the $i^{\text{th}}$ vertex, and $1\left(z_{i}=z_{j}\right)$ is the indicator function taking value 1 if $z_{i}=z_{j};0$ otherwise. A high value of modularity indicates that the network is assortative.

### Variational Information Distance

The Variational Information (VI) distance (Meilă, 2007) measures the quality of two community assignments $C^{\left(1\right)}$ and $C^{\left(2\right)}$ based on entropy. For any community assignment C, we define the entropy of the assignment as

$$H(C)=-\sum_{1\leq k\leq K}P(k)\;\text{log}\;P(k),$$

where $P(k)=\frac{n_{k}}{n}$ is the proportion of vertices being assigned to $k^{\text{th}}$ community for 1≤k≤K. Now, the mutual information for two community assignments $C^{\left(1\right)}$ and $C^{\left(2\right)}$ is defined in terms of Kullback-Leibler divergence as follows,

$$I\left(C^{\left(1\right)},C^{\left(2\right)}\right)=\sum_{1\leq k_{1}\leq K}\sum_{1\leq k_{2}\leq K}P\left(k_{1},k_{2}\right)\;\text{log}\frac{P\left(k_{1},k_{2}\right)}{P\left(k_{1}\right)P\left(k_{2}\right)},$$

where $P\left(k_{1},k_{2}\right)=\frac{\left|c_{k_{1}}^{\left(1\right)}\cap c_{k_{2}}^{\left(2\right)}\right|}{n}$ calculates the proportion of vertices which are common in two different community assignments. Thus, the VI distance is defined as

(8) $$VI\left(C^{\left(1\right)},C^{\left(2\right)}\right)=H\left(C^{\left(1\right)}\right)+H\left(C^{\left(2\right)}\right)-I\left(C^{\left(1\right)},C^{\left(2\right)}\right).$$

A low value of VI distance indicates that the community assignments convey similar information.

### Calinski-Harabasz Index

The Clainski-Harabasz (CH) index (Calinski & Harabasz, 1974) measures strength of dissimilarity within-group and among-group sum of squares. For a community assignment C, it is defined as pseudo-F ratio,

(9) CH(K)=SSA(K−1)SSW(n−K),

where the within-group sum of squares is defined as

$$\text{SSW}=\sum_{1\leq k\leq K}\frac{1}{n_{k}}\sum_{i,j\in C_{k},i<j}d_{ij}^{2},$$

and the among-group sum of squares is defined as SSA=SST-SSW with

$$\text{SST}=\frac{1}{n}\sum_{1\leq i<j\leq n}\;d_{ij}^{2}.$$

It is evident that the value of the index will always be positive. If the ratio is very large that indicates high separateness and compactness (Milligan & Cooper, 1985).

### C-Index

The C-index (Milligan & Cooper, 1985) measures the within-group distance and computed as,

(10) $$C\left(k\right)=\frac{SW-S_{\text{min}}}{S_{\text{max}}-S_{\text{min}}},$$

where the sum of within-community distances

$$SW=\sum_{1\leq k\leq K}\;\sum_{i,j\in C_{k},i<j}\;d_{ij}.$$

$S_{\text{min}}$ and $S_{\text{max}}$ is the sum of the $N_{W}$ smallest and largest distances respectively, where NW=∑1≤k≤Knk2 is the total number of vertices is the same community. C-index lies between [0,1] and small value suggests optimal number of communities.

### Dunn Index

The Dunn index (Dunn, 1973) also measures the within-group distance and is defined as,

(11) $$D(K)=\frac{\underset{1\leq r<s\leq K}{\text{min}}\;\delta \left(C_{r},C_{s}\right)}{\underset{1\leq k\leq K}{\text{max}}\;\Delta_{k}},$$

where $\delta \left(C_{r},C_{s}\right)$ is the inter-group distance between communities $C_{r}$ and $C_{s}$, is defined as $\delta \left(C_{r},C_{s}\right)=\underset{i\in C_{r},j\in C_{s}}{\text{min}}\;d_{ij}$. In the denominator, the diameter of community $C_{k}$ is defined as $\Delta_{K}=\underset{i,j\in C_{k}}{\text{max}}\;d_{ij}$. The index is maximized when clusters are compact in other words the inter-group distance is large, and the diameter is small.

### Consensus algorithm

In this section, we outline the algorithm for obtaining a consensus solution given a fixed community size. Since the community labels generated in two independent iterations may not align, we first rearrange the community labels using the Hamming distance (Hamming, 1950). Let $z=\left(z_{1},z_{2},\ldots ,z_{n}\right)$ and $z^{\prime }=\left(z_{1}^{\prime },z_{2}^{\prime },\ldots ,z_{n}^{\prime }\right)$ denote the estimated community labels from two independent iterations with community size K. Define, ${𝒢}_{K}=\{\pi :\{1,2,\ldots ,K\}\mapsto \{1,2,\ldots ,K\}\}$ as the set of all possible permutation of {1,2,…,K}. The Hamming distance between the community assignments z and $z^{\prime }$ is given by

(12) $$d_{{𝒢}_{K}}\left(z,z^{\prime }\right)=\underset{\pi \in {𝒢}_{K}}{\text{min}}\;\frac{1}{n}\sum_{i=1}^{n}\;1\left(z_{i}\neq \pi \left(z_{i}^{\prime }\right)\right).$$

In essence, the Hamming distance measures the minimum average proportion of mismatches between z and $z^{\prime }$ across all possible community relabeling of $z^{\prime }$. The minimization problem in equation (12) has a complexity of $O\left(n+K^{3}\right)$ using the Hungarian algorithm (see (Kuhn, 1955) for more details). The consensus algorithm is presented as follows:

- Step 1: Select a community assignment from the set of all independent solutions to serve as the reference label.
- Step 2: Compute the Hamming distance between the reference label and each of the remaining solutions.
- Step 3: For each solution, determine the permutation mapping π that minimizes the optimization problem described in equation (12). The communities are relabeled accordingly using the minimizer π.
- Step 4: Finally, each ROI is assigned to the community obtained as the minimizer in Step 3 appearing most frequently across the relabeled solutions and thus forming the consensus community assignment.

This algorithm resolves label-switching ambiguities, resulting in a stable and interpretable community structure. The final community assignment is equivalent to the original one up to a permutation of class labels, so the number of detected communities is preserved. Importantly, the method does not depend on any underlying community structure. Upon aggregation of information across multiple solutions, the consensus algorithm provides a more robust and reliable estimate of the true underlying communities.

## Results

### Simulations

**Figure 2:**
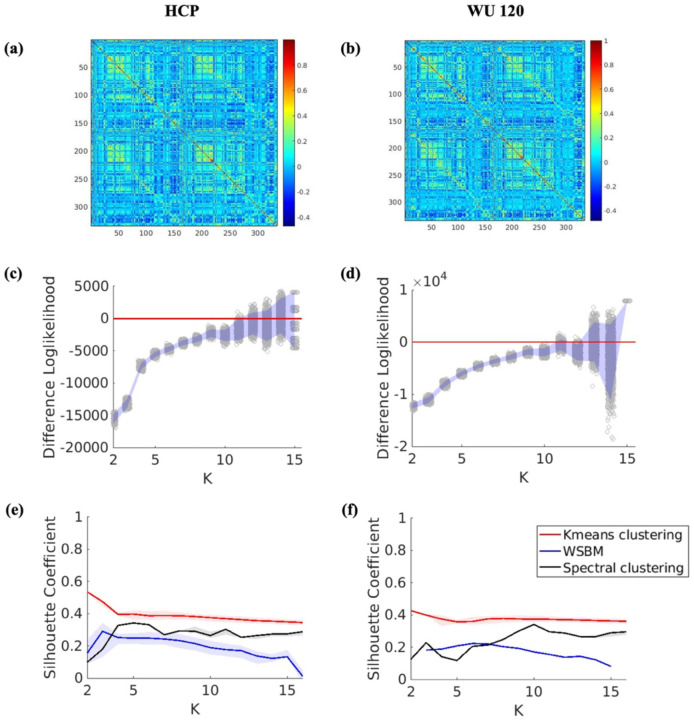
**(a)** Average FC matrix for HCP data. **(b)** Average FC matrix for WU 120 data. **(c,d)** Bootstrap log-likelihood difference plot of the solutions from WSBM for HCP and WU 120 data receptively. The red line corresponds to the null value zero. **(e,f)** Silhouette coefficient values with interquartile range for HCP and WU 120 data respectively.

The community detection algorithms were compared using various methods, with model parameters systematically varied to simulate real-life networks and K=5 communities were simulated. For WSBM, both the difference likelihood plot and silhouette coefficient measure identified five communities, which matched the oracle number of communities. The silhouette coefficient plot for the spectral clustering method also detected five communities, whereas K-means identified four (Fig. 1.d). For WSBM, community labels were assigned based on the maximum log-likelihood from the valid community assignments (Fig. 1.e). In contrast, for spectral clustering and K-means clustering, there were no established post hoc evaluation metrics for determining the optimal number of communities (Figs. 1.c and 1.d). Although the silhouette coefficient failed to consistently identify the correct number of communities, it had been widely applied to estimate the final community labels for these clustering methods in community detection (Craddock et al., 2012; Ryali et al., 2015; Tooley et al., 2022; Tu et al., 2024). For this reason, we adopted the silhouette coefficient to determine the final community assignments. Accordingly, the community assignment corresponding to the highest silhouette coefficient across independent replications was treated as the final solution. We also examined the effect of alternative post hoc evaluation measures on real data (see Figs. S1 – S3); however, these methods similarly failed to identify the optimal number of communities. Despite detecting different numbers of communities with the silhouette coefficient, the high similarity measures, such as NMI, and the low Hamming distance values across 1000 replications demonstrated that all three algorithms exhibited comparable performance in terms of stability (Figs. 2.i and 2.j). Spectral clustering displayed higher stability as it has the lowest hamming distance and highest NMI; nevertheless, each clustering methods produced similar solutions up to a rearrangement. This result can be attributed to the greater stability of spectral clustering compared with K-means, particularly when the true communities are not linearly separable in the original Euclidean space but become linearly separable in the eigenspace of the graph Laplacian.

### Comparisons between methods applied to real data

#### Adult Datasets

**Figure 3:**
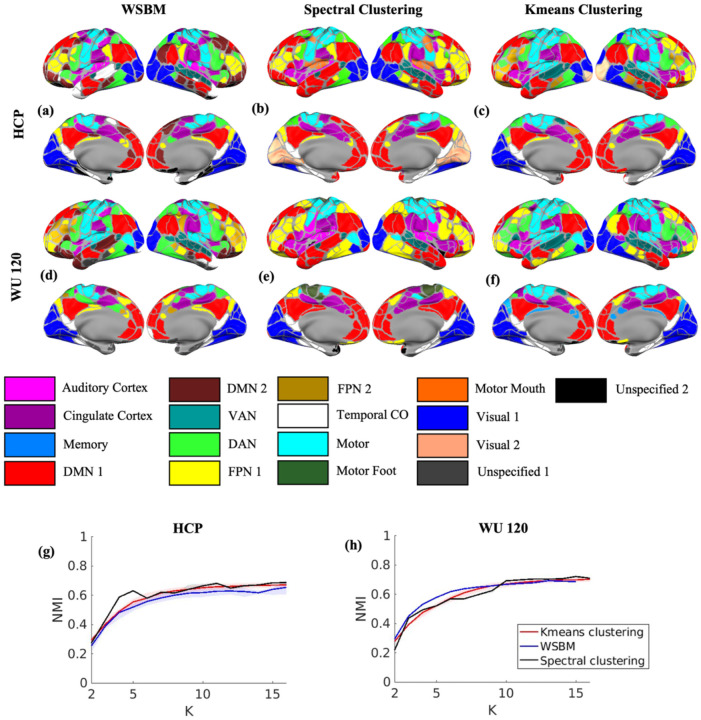
**(a,d)** Brain surface plot of the solution with highest log-likelihood from WSBM for K=11 for HCP and WU 120 data respectively. **(b,e)** Brain surface plot of the solution with highest silhouette coefficient from spectral clustering for K=11 for HCP and WU 120 data respectively. **(c,f)** Brain surface plot of the solution with silhouette coefficient from K-means clustering for K=11 for HCP and WU 120 data respectively. **(g,h)** NMI values with interquartile range between the community assignment solutions from three methods and Gordon networks for HCP and WU 120 data respectively.

For all three algorithms, the silhouette coefficient was unable to determine the optimal number of communities and did not exhibit any global maximum for either the HCP or WU 120 adult datasets (Figs. 2.e and 2.f). The difference log-likelihood plot, based on 2000 bootstrap iterations, highlighted K=11,12,13,and14 as plausible solutions. We opted for K=11, as it represents the smallest K for which the 95% bootstrap confidence interval for the difference log-likelihood includes the null value of zero (Figs. 2.c and 2.d). For generating the brain plots (Figs. 3.a–3.f), we use K=11 as the optimal number of communities, determined from the bootstrap difference log-likelihood plot. For this fixed value of K, the solution with the highest log-likelihood among all valid solutions is chosen as the final solution for WSBM. On the other hand, for Spectral Clustering and K-means, the solution with the highest silhouette coefficient for K=11 is selected as the final solution, based on 1000 independent replications. Although the silhouette coefficient did not yield an optimal solution for the number of communities, we used it to select the final solution for each K due to its widespread use in the community detection literature, as discussed in previous sections. The brain plot analysis for all three algorithms further showed that, for both datasets, the NMI values were similar across the algorithms when using the Gordon networks (Gordon et al., 2016). Notably, for the numbers of communities greater than 10, the NMI values were moderately high (> 0.6) (Figs. 3.g and 3.h).

**Figure 4:**
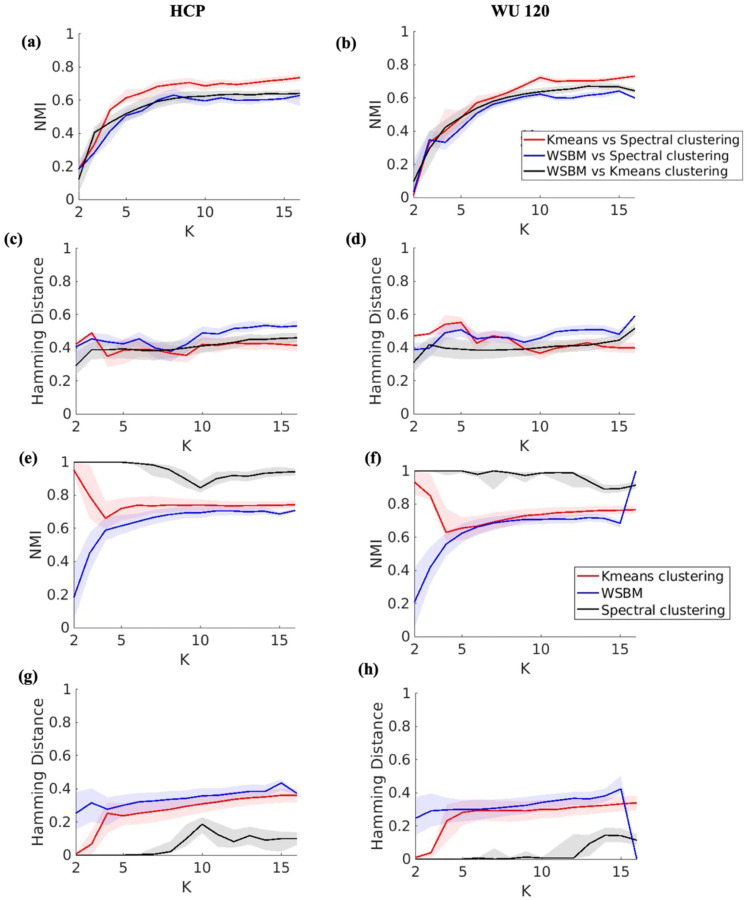
**(a,c)** NMI and Hamming distance values and the interquartile range for all pairwise community assignments between different clustering algorithms on HCP data. **(b,d)** NMI and Hamming distance values and the inter quartile range for all pairwise community assignments between different clustering algorithms on WU 120 data. **(e,g)** NMI and Hamming distance values with interquartile range for HCP data respectively. **(f,h)** NMI and Hamming distance values with interquartile range for WU 120 data respectively.

For the HCP and WU 120 datasets, the spectral clustering algorithm yielded very high NMI values (> 0.8) across the solutions from the independent iterations. In contrast, the NMI values for K-means clustering and WSBM are moderately high (between 0.6 and 0.8) when the number of communities exceeded 5 (Figs. 4.e and 4.f). Hamming distance followed a similar pattern, with spectral clustering showing the lowest values (between 0 and 0.2), while K-means and WSBM had moderately low values (between 0.2 and 0.4) (Figs. 4.g and 4.h). The reproducibility analysis demonstrated that for the HCP and WU 120 datasets, the three algorithms produced moderately high NMI values (> 0.6) when the number of communities exceeded 7. For community numbers between 4 and 7 (inclusive), both K-means and Spectral Clustering showed strong agreement, with moderately high NMI values, while WSBM exhibited moderate agreement with K-means and Spectral Clustering, registering low NMI values (<0.4) (Figs. 4.a and 4.b). The stability analysis revealed that the Hamming distance values were generally low (0.3–0.5) across most K values, indicating strong agreement among the three clustering algorithms (Figs. 4.c and 4.d). We also used several post hoc measures as discussed in the Methods Section to estimate the number of communities for the two adult datasets. However, none of the post hoc measures identified a clear global optimum (Figs. S1 and S2).

Overall, all three clustering algorithms generally recovered the sensory and association networks (Uddin et al., 2019) and the brain plots from the consensus algorithm of the K-means and spectral clustering results (Figs. S3 and S4) were generally similar to the results from WSBM based on the number of communities derived from the highest log-likelihood plot. Figs. S4.a–S4.f present the FC matrices selected based on the highest log-likelihood (WSBM) and silhouette index (Spectral Clustering and K means).

#### Infant Datasets

**Figure 5:**
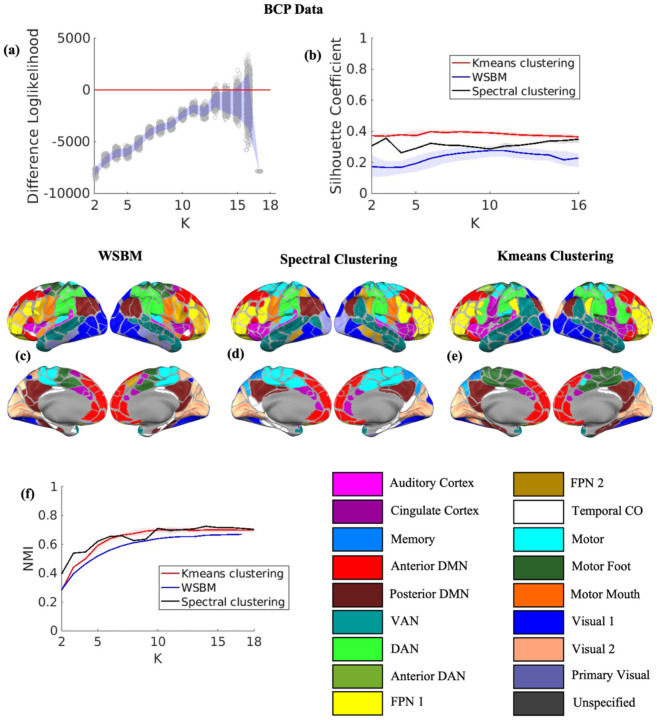
**(a)** Bootstrap log-likelihood difference plot of the solutions from WSBM for BCP data. The red line corresponds to the null value zero. **(b)** Silhouette coefficient values with interquartile range for BCP data. **(c)** Brain surface plot of the solution with highest log-likelihood from WSBM for K=15 for BCP data. **(d)** Brain surface plot of the solution with highest silhouette coefficient from spectral clustering for K=15 for BCP data. **(e)** Brain surface plot of the solution with silhouette coefficient from K-means clustering for K=15 for BCP data. **(f)** NMI values with interquartile range between the community assignment solutions from three methods and Kardan atlas for BCP data.

For the BCP dataset, the difference in log-likelihood plot, derived from 2000 bootstrap iterations, suggested that the optimal number of communities is K=15 (Fig. 5.a). The silhouette coefficient was unable to determine the optimal number of communities for any of the three algorithms and, akin to the adult datasets, lacked a distinguishable global maximum (Fig. 5.b). Similar brain systems were observed across the community detection methods (Figs. 5.c–5.e). While WSBM was able to identify motor hand, foot, and mouth, spectral clustering was only able to recover dorsal and ventral motor systems while K-means was only able to recover hand and foot. Communities including the DMN and FPN were consistently split into anterior and posterior components of their adult network counterparts across all three methods. The NMI values were moderately high when compared to the Kardan baby network atlas (Kardan et al., 2022). Figs. S4.g–i shows the FC matrix plots and Figs. S5.g–S5.i shows the axial view of the baby connectomes.

#### Consensus results for all datasets

Although we used the minimum value of K as the main criterion for selecting the optimal number of communities, the bootstrap differences in log-likelihood (Figs. 3.c, 3.d and 6.a) indicated that alternative choices of K were also plausible. Therefore, we evaluated consensus communities for all values of K where the null value 0 fell within the range of the bootstrap log-likelihood differences. To construct the consensus, we applied the algorithm described previously in the section on Statistical Analysis to all valid WSBM solutions for each fixed K. As reference communities, we chose the solution with the highest log-likelihood for that K. The brain surface plots of the consensus solutions for the HCP, WU 120, and BCP datasets are shown in Figs. S6.a–S6.c, S7.a–S7.c, and S8.a–S8.d, respectively.

When comparing the consensus solutions with the highest log-likelihood and silhouette coefficient solutions for adults (Figs. 3.a–3.f), the results were consistent at K=11. In contrast, for the infant dataset (Figs. 5c–5.e), the consensus solution diverged, with the posterior DMN and VAN combined into a single network—highlighting developmental differences in network organization. For each fixed K, we also plotted the rearranged community labels across independent iterations, grouping them by community labels for the HCP, WU 120, and BCP datasets (Figs. S6.d–S6.f, S7.d–S7.f, S8.e–S8.h). To visualize label changes across valid solutions, we followed the ordering of the Gordon atlas (Gordon et al., 2016). These findings underscore both the stability of the consensus procedure and its ability to yield interpretable community structures under multiple candidate values of K.

Finally, we examined the functional connectivity (FC) matrices averaged across subjects to assess community-level connectivity strength. The averaged FC matrices, grouped by community labels, are shown in Figs. S9.a–S9.j. These plots reveal non-assortative structures in the human brain, consistent with the patterns observed in Figs. S4.a–S4.f for the highest log-likelihood and silhouette coefficient solutions.

## Discussion

In this article, we addressed the problem of determining the optimal number of communities for adult and infant brain networks via three clustering algorithms viz. WSBM, Spectral clustering, and K-means. Using a bootstrap-based difference log-likelihood approach, we found that the brain connectome optimally comprised 11 communities for adults and 15 communities for infants. However, post hoc community evaluation metrics failed to detect an optimal number of communities for spectral clustering and K-means clustering. Our results show that the WSBM community solution for the adult brain network resembles the Gordon networks (Gordon et al., 2016). Our results provide additional evidence that the community solution for the infant brain differs from the adult brain community structure building on prior work which used the Infomap algorithm (Kardan et al., 2022; Marrus et al., 2018; Tu et al., 2024).

We evaluated the performance of the clustering algorithms on the synthetic data. All three clustering methods exhibited similar performance on the synthetic data, having similar NMI and Hamming distance which ensures the reproducibility of these algorithms. The most popular post hoc measure, silhouette coefficient, was able identify the optimal number of communities only for WSBM and Spectral Clustering. It incorrectly identifies four as the optimal number of communities for K-means clustering. We also performed similar analyses on adult and infant data with various metrics, viz. CH-index, C-index, modularity, and VI distance where, despite their known utility in the literature for revealing unknown communities, none of these metrics delivered valid solutions in our context, similar to an observation made in Tooley et al. (2022). A potential reason for this may be that these metrics perform well for an assortative structure in the community solution, which can be achieved by thresholding the FC matrix. Our analysis does not assume a predefined network structure and instead performs clustering directly on the complete FC matrix. Thus, the bootstrap-based log-likelihood difference for WSBM has been used to determine the optimal number of communities for the real-life data sets which has shown to successfully recover the optimal number of communities for both synthetic and real-life datasets.

Our analysis using WSBM identified 11 communities in both HCP and WU 120 adult datasets. Notably, all three clustering algorithms exhibited similar community solutions. Specifically, the solutions from K-means clustering and spectral clustering had the highest NMI and lowest hamming distance values for all choices of K. This indicates that K-means and spectral clustering produce similar community assignments. Furthermore, all three methods had high (or moderately high) NMI values and low hamming distance when the number of communities is greater than 5, indicating that the algorithms are highly reproducible. DMN and Motor networks appear to be consistent across all three clustering algorithms. WSBM treats Visual 1 & 2 as the same network consistent with Gordon et al. (2016) while the other two methods split it into two different regions (Gordon et al., 2017). The optimal solutions also aligned closely with the Infomap solution (Gordon et al., 2016) as they have moderately high NMI values with the Gordon atlas. Figs. 4.a–4.h also shows that all three algorithms can detect the major community structure (DMN, Auditory Cortex, Motor, FPN, Visual, etc.) of the adult brain network. The latter is also in agreement with the consensus plots that show that the communities are consistent across independent iterations for different choices of K.

For the infant brain, WSBM was able to identify 15 communities. Similar to the adult datasets, all three methods produced comparable clustering solutions for different values of K. Analysis on the BCP data suggested that infant brain communities have more variability than adult brain communities. Nevertheless, the solutions effectively captured the major community structure of the brain. Additionally, for the infant brain, the DMN and FPN are divided into two distinct communities with anterior and posterior components. Consensus results also confirmed that community assignments remain consistent across different choices of K. Optimal community assignments also have moderately high NMI values with previously published the Infomap solutions in the infant/toddler age (Eggebrecht et al., 2017; Kardan et al., 2022; Tu et al., 2025). While Infomap solutions for neonate and toddler datasets can be used to identify more ‘adult like’ networks by increasing the density of the graph (Sylvester et al., 2023; Tu et al., 2025), these long-range connections are weakly connected at these ages. The latter findings may be explained by early stages of brain development or by the fact that the scans were acquired during sleep. Here we demonstrate, using the negative log likelihood from the WSBM model that the optimal number of communities splits several higher order association networks into anterior and posterior components in young children.

This article provides some insight into selecting the optimal number of communities for adult and infant brain connectomes. Our analysis suggests that different types of post hoc community evaluation metrics may fail to detect the optimal number of communities. Here we demonstrate that WSBM, with the help of difference log-likelihood, is an effective method to obtain the unknown number of communities. Furthermore, we illustrate the fact that the community structure of the infant brain is distinctly different from that of the adult brain, indicating the need for developing separate atlases for infant data. However, one needs to keep in mind that some of the community detection algorithms mentioned above (including WSBM) are affected by detectability issues and resolution limits, which can impact their performance. The detectability limit is known to depend on the graph’s characteristics rather than the specific algorithm used (Reichardt & Leone, 2008). Additionally, the WSBM is subject to a resolution issue. For a network of size n, the maximum number of detectable communities is $\sqrt{n}$ when using maximum log-likelihood estimation (Choi et al., 2012; Peixoto, 2013). As discussed earlier, the community assignments in WSBM are based on the maximum posterior assignment probability. However, this does not automatically guarantee that the number of estimated communities will match the desired number specified as input (Faskowitz & Sporns, 2020). It is also worth mentioning that other widely used community detection algorithms, such as modularity maximization and Infomap, encounter similar issues due to thresholding. In sparse graphs, thresholding may remove edges, leading to multiple singleton communities. Finally, from a computational standpoint, while these methods are scalable to higher-resolution networks, their scalability can vary significantly depending on the algorithm.

## Supplementary Material

Supplement 1

Supporting information for this article is available in the Supplementary Material. Human Connectome Project data is available at https://db.humanconnectome.org/. WashU 120 data is available at https://openneuro.org/datasets/ds000243/versions/00001. Baby Connectome data is available at https://nda.nih.gov/edit_collection.html?id=2848. The code for reproducibility of these results can be found in https://github.com/ayoushmanb/BrainNetworks_WSBM_KM_SC.

## Figures and Tables

**Table 1:** Runtime complexity of algorithms

Algorithm	Time complexity
WSBM	$O\left(n^{2}K^{2}\right)$
Spectral Clustering	O(n+K)
K-means Clustering	O(n+K)
