# Supplementary material for: Comparative Evaluation of Assumption Lean Community Detection Methods for Human Connectome Networks
Source: bioRxiv. 2025 Nov 14:2025.11.13.688333. Preprint. [Version 1] doi: 10.1101/2025.11.13.688333 (PMC12642645; doi:10.1101/2025.11.13.688333)
Supplement: Supplement 1 [file media-1.pdf]

Supplement to "Comparative Evaluation of Assumption Lean Community Detection Methods for Human Connectome Networks"

HCP Data

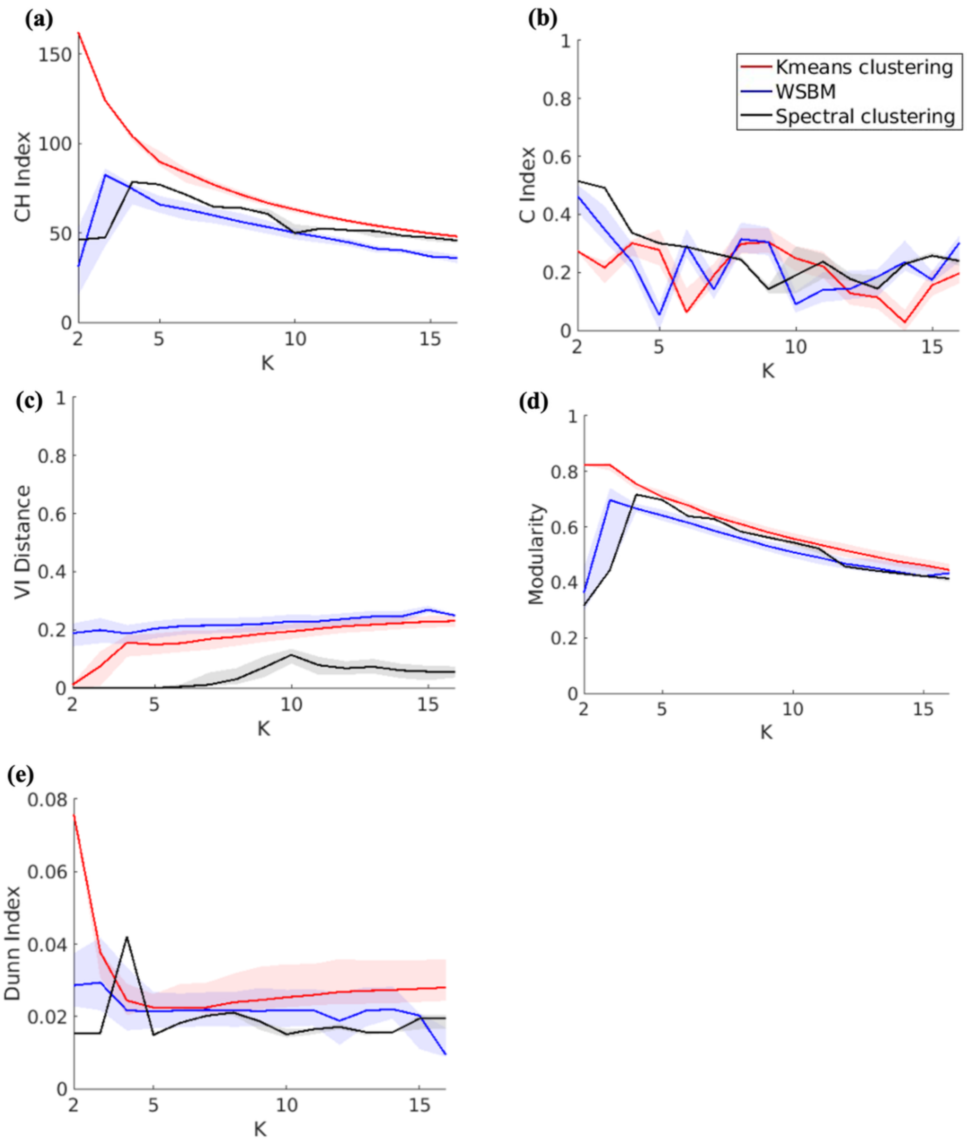

Figure S1: Evaluation of different measures of clustering solutions for HCP data. **(a)** CH index, **(b)** C-index, **(c)** VI Distance, **(d)** Modularity, **(e)** Dunn Index.

WU 120 Data

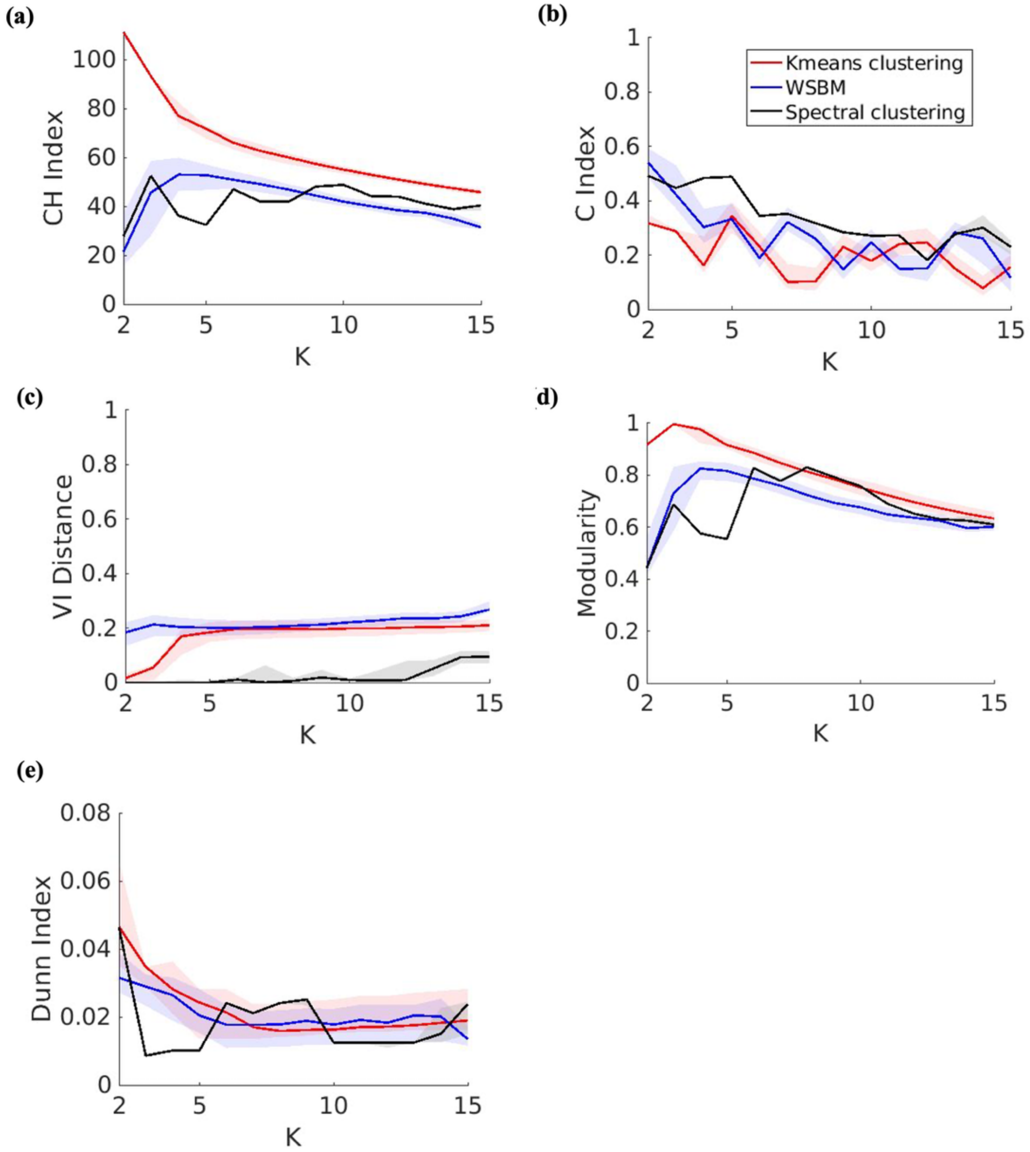

Figure S2: Evaluation of different measures of clustering solutions for WU 120 data. **(a)** CH index, **(b)** C-index, **(c)** VI Distance, **(d)** Modularity, **(e)** Dunn Index.

BCP Data

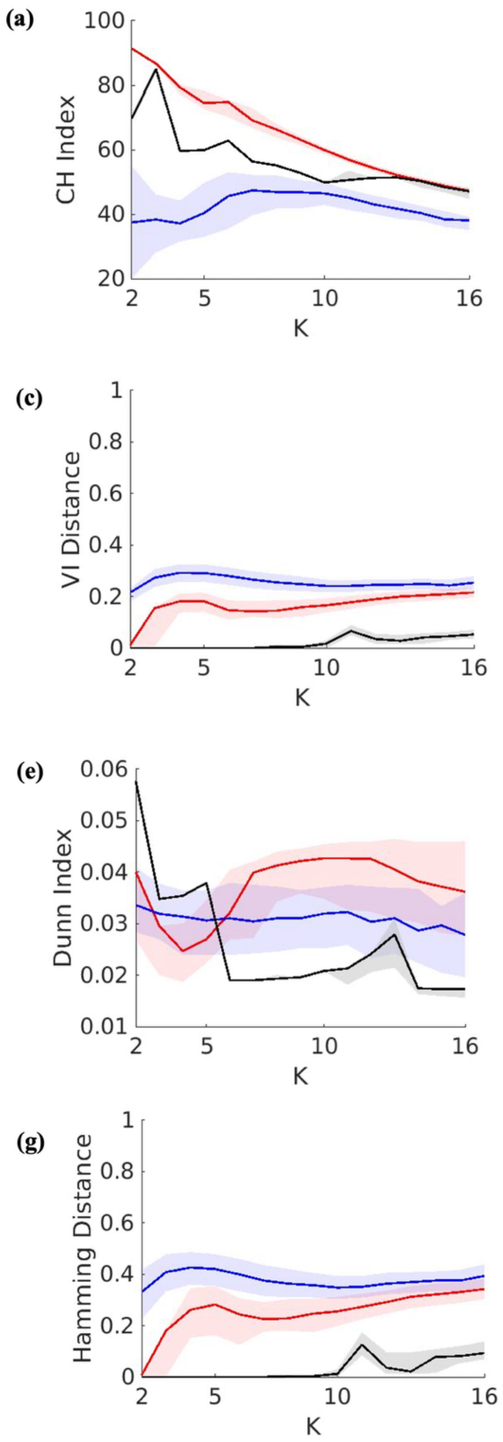

Figure S3: Evaluation of different measures of clustering solutions for BCP data. **(a)** CH index, **(b)** C-index, **(c)** VI Distance, **(d)** Modularity, **(e)** Dunn Index, **(f)** NMI, **(g)** Hamming Distance.

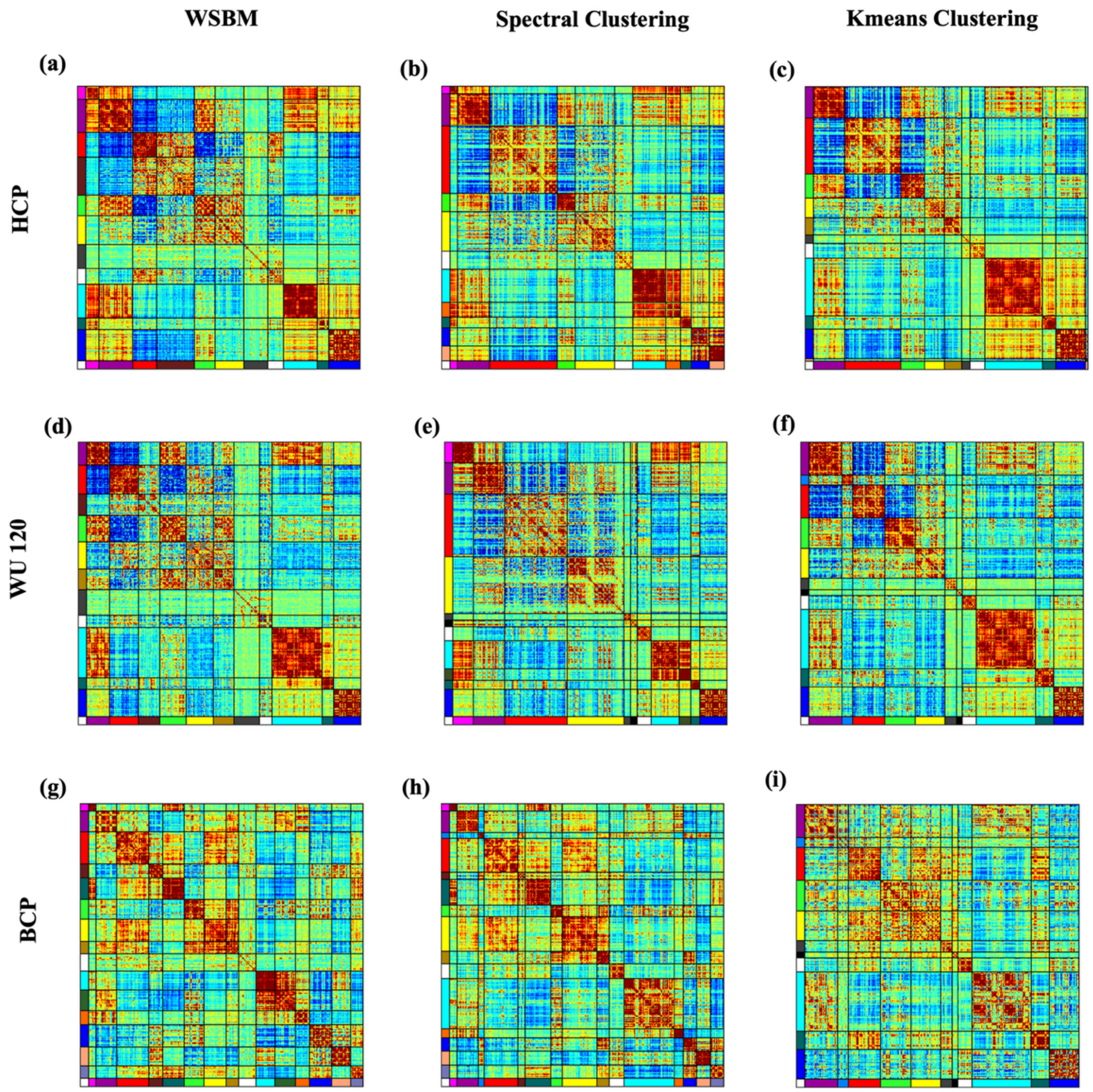

Figure S4: (a-f) Average Fc matrix plots for adult data with 11 communities. **(g-i)** Average Fc matrix plots for baby data with 15 communities.

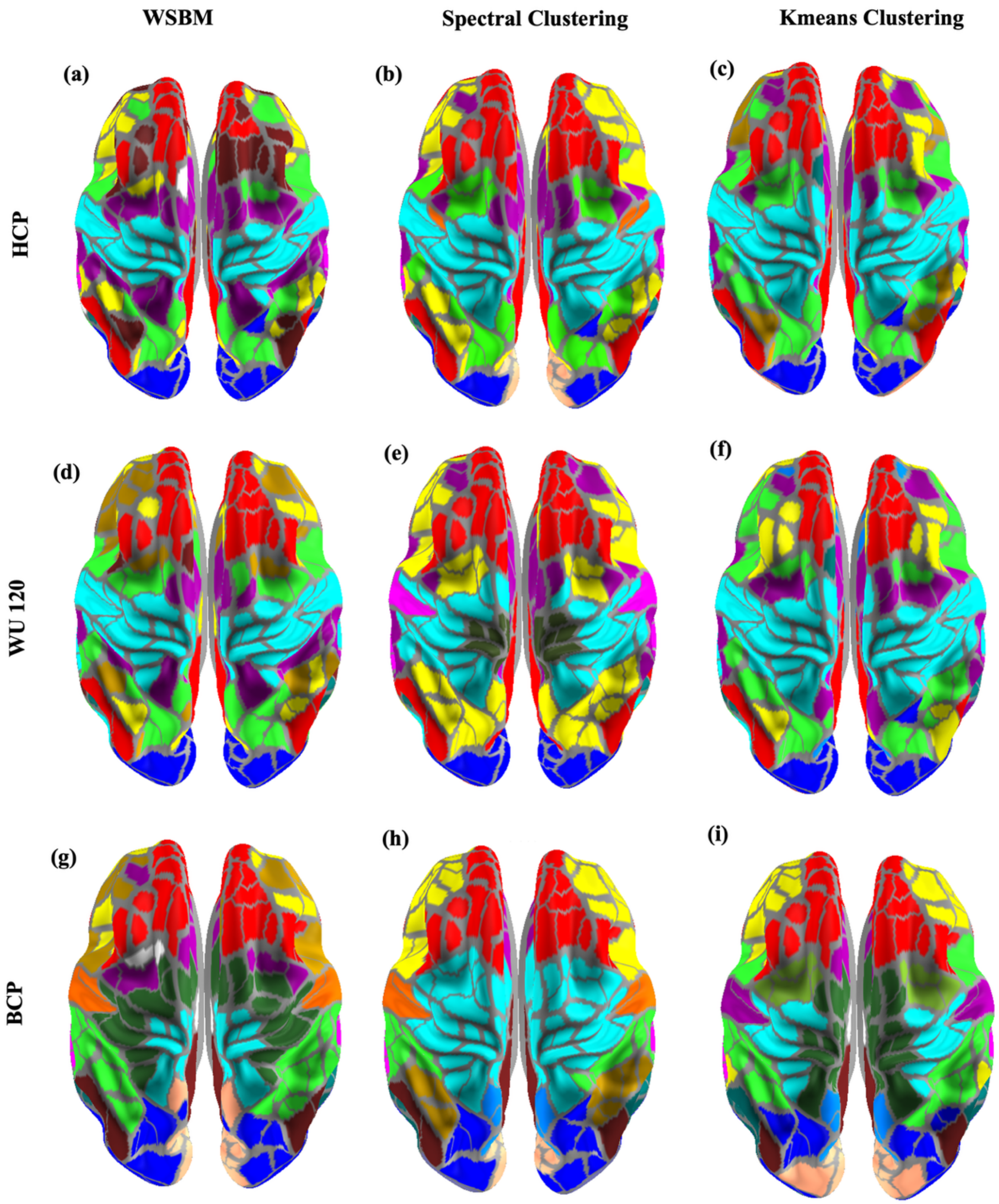

Figure S5: (a-f) Brain Surface plots for adult data with 11 communities. **(g-i)** Brain surface plots baby data with 15 communities.

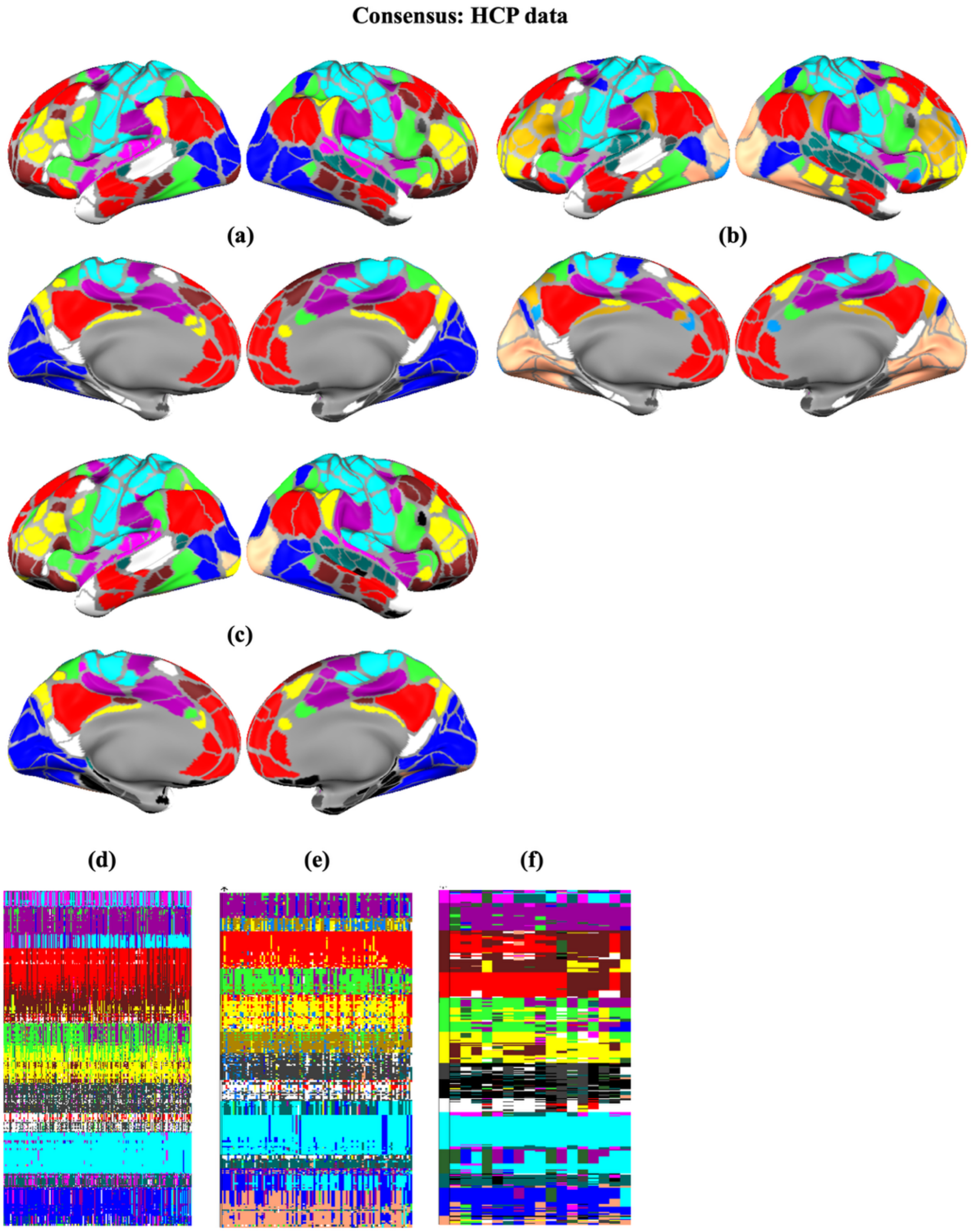

Figure S6: (a-c) Brain Surface plots of consensus for WSBM on HCP data with K = 11,12, and 14 respectively. **(d-f)** Community assignments for valid replications of WSBM on HCP data with K = 11,12, and 14 respectively.

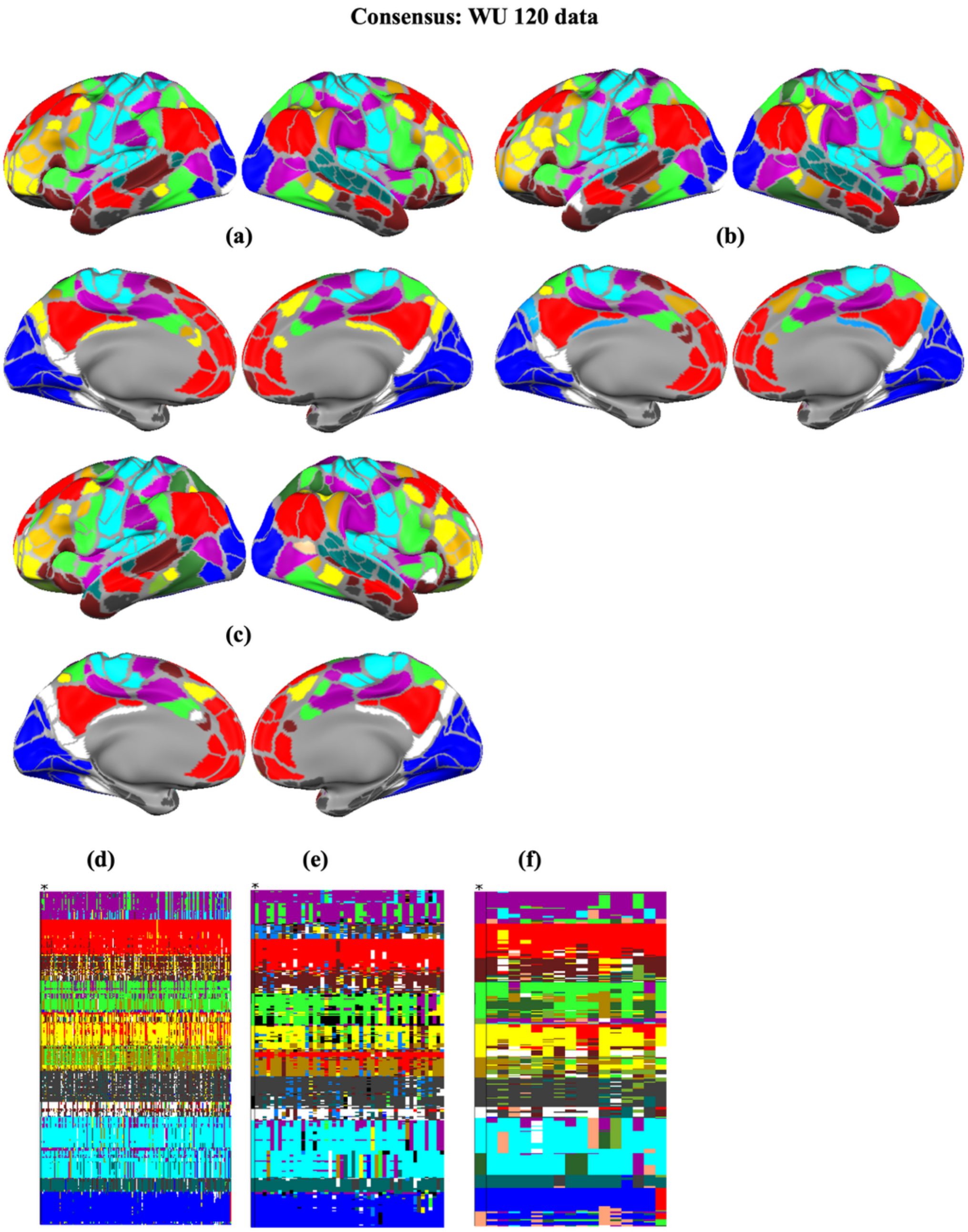

Figure S7: (a-c) Brain Surface plots of consensus for WSBM on WashU 120 data with K = 11,13 and 14 respectively. **(d-f)** Community assignments for valid replications of WSBM on WashU 120 data with K = 11,13 and 14 respectively.

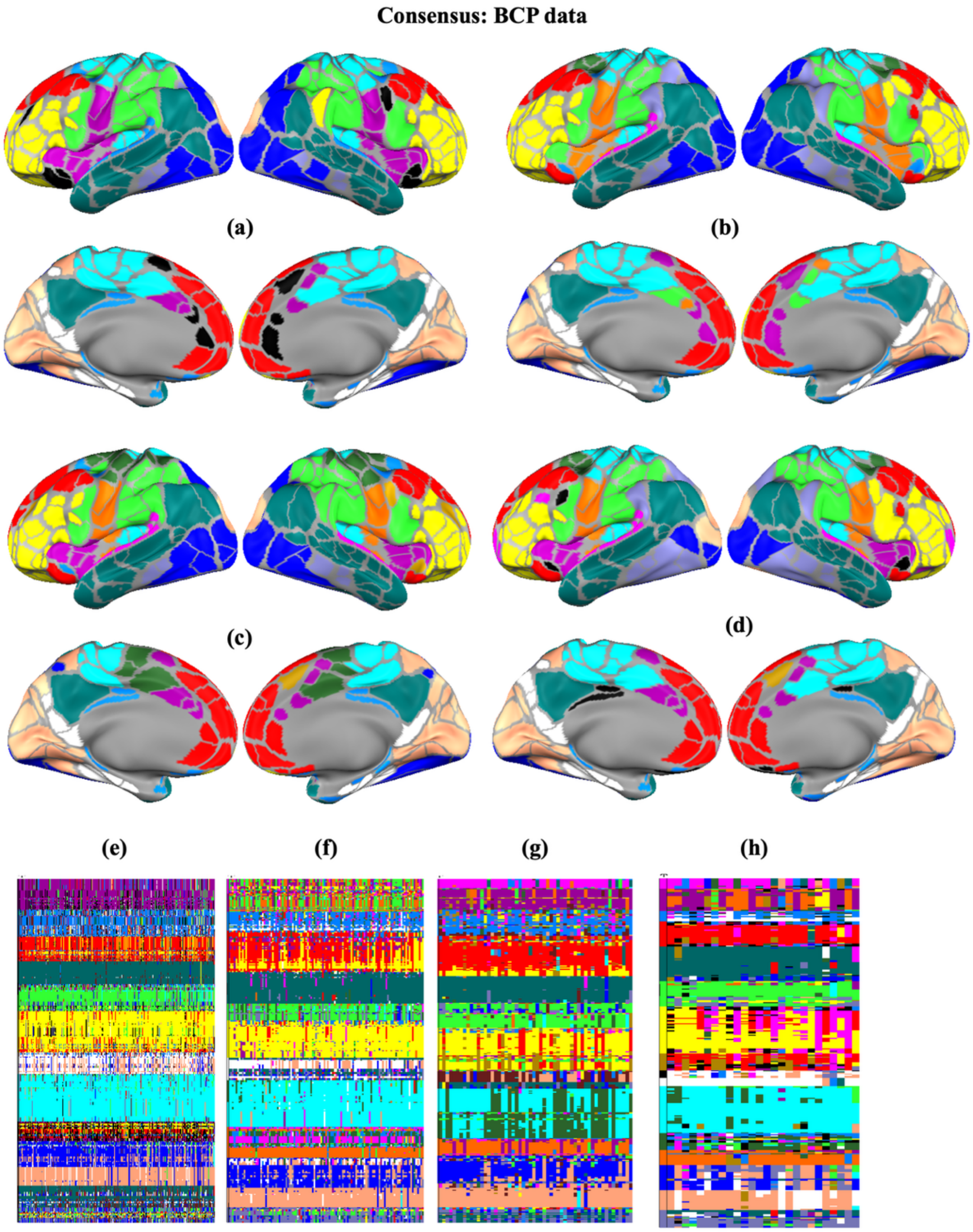

Figure S8: (a-d) Brain Surface plots of consensus for WSBM on BCP data with $K = 13,14,15$ and 16 respectively. **(e-f)** Community assignments for valid replications of WSBM on BCP data with $K = 13,14,15$ and 16 respectively.

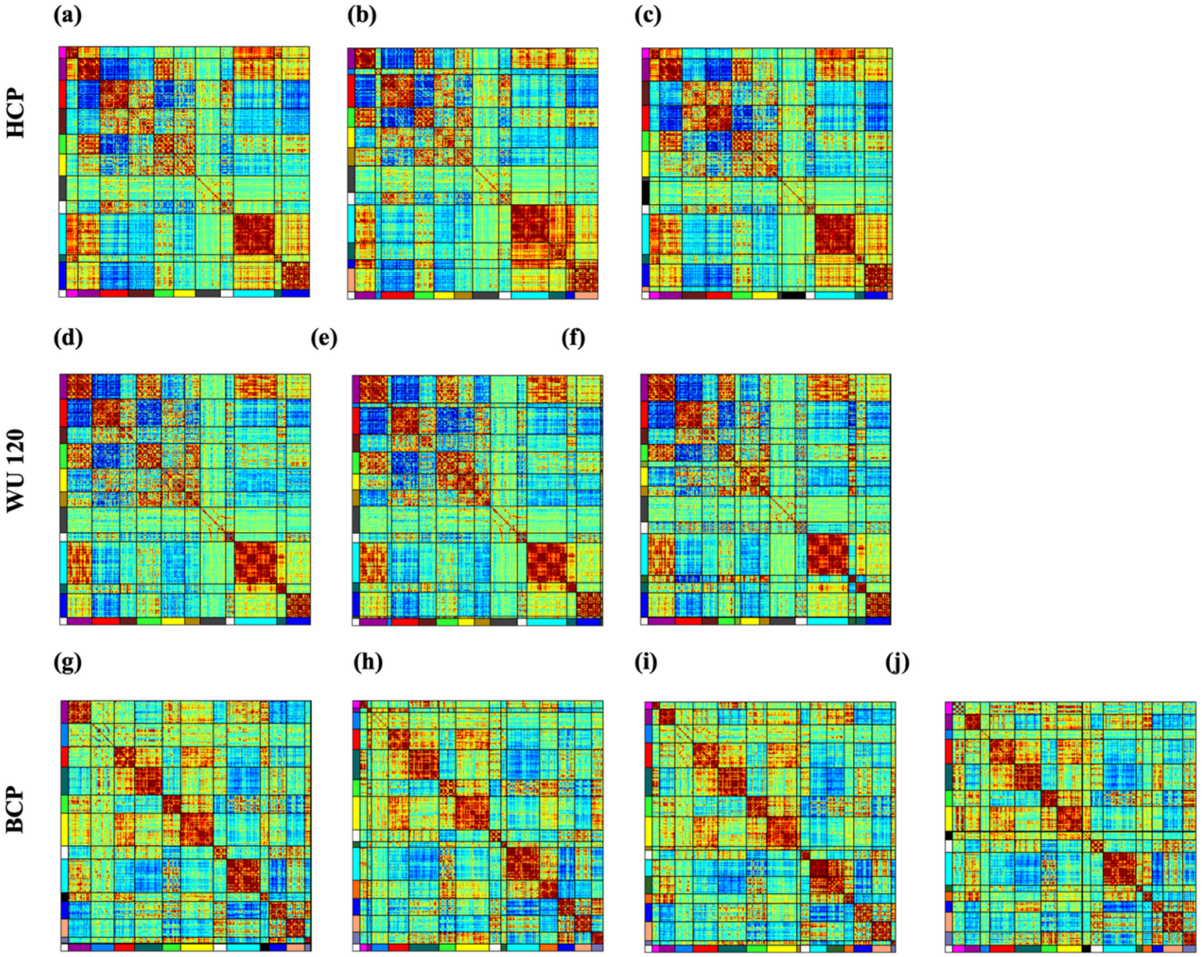

Figure S9: (a-c) Average FC matrix plots of consensus for WSBM on HCP data with K = 11,12 and 14 respectively. **(d-f)** Average FC matrix plots of consensus for WSBM on WashU 120 data with K = 11,13 and 14 respectively. **(g-j)** Average FC matrix plots of consensus for WSBM on BCP data with K = 13,14,15 and 16 respectively.
